# Concurrent chemotherapy in oropharyngeal cancer: Cisplatin wins

**DOI:** 10.18632/oncotarget.26594

**Published:** 2019-01-18

**Authors:** Arya Amini, Sana D. Karam

**Affiliations:** Department of Radiation Oncology, City of Hope Cancer Center, Duarte, CA, USA

**Keywords:** oropharynx, concurrent chemoradiotherapy, cisplatin, carboplatin, cetuximab

Rates of oropharyngeal squamous cell carcinoma (OPSCC) appear to be increasing over the past decade, much due to the rise in the prevalence of human papillomavirus (HPV) related OPSCC [1]. The addition of concurrent chemotherapy with cisplatin (CDDP) to RT (CRT) is the standard of care for OPSCC patients opting for an organ-preservation approach, based upon an improvement in overall survival (OS) with the added cost of increased potential for short- and long-term morbidity observed in multiple prospective randomized trials [2]. Additional options suggested by the National Comprehensive Cancer Network (NCCN) guidelines for those who are unable to tolerate high-dose CDDP include cetuximab (CTX) and carboplatin [3]. Prior to this past year, there were only limited small retrospective series comparing standard of care CDDP to CTX or carboplatin. Recently, three studies published, two of which are prospective randomized controlled trials, have provided greater insight on this topic [4–6].

Using the linked Surveillance, Epidemiology and End Results (SEER)-Medicare database, our group recently compared OS, toxicity, and total treatment costs between concurrent CTX, carboplatin, and CDDP in patients with OPSCC undergoing definitive CRT. A total of 409 patients were evaluated; 167 (41%) received concurrent CDDP, 173 (42%) concurrent CTX, and 69 (17%) received concurrent carboplatin. Oropharynx sites included base of tongue (55%), tonsil (32%), and oropharynx not otherwise specified (14%). When compared to concurrent CDDP, 2-year OS was lower with CTX (HR, 1.68; *p =* 0.020) but no different in comparison to carboplatin (HR, 1.31; *p =* 0.362). Higher OS rates came at a risk of higher toxicity with concurrent CDDP, including greater antiemetic use and hospital visits for nausea/emesis, diarrhea or dehydration. Pneumonia rates were found to be higher with carboplatin use. In regards to total treatment costs, corrected mean per patient spending for CTX and carboplatin were significantly higher than CDDP ($61,133 and $65,721 *vs* $48,709). Perhaps not surprising, given the concern for higher rates of toxicity with concurrent CDDP, those who were older or with multiple comorbidities were less likely to receive CDDP. Our population-based analysis demonstrated CDDP appeared to be associated with the highest OS, followed by carboplatin and CTX. Treatment costs were also lower with concurrent CDDP. Acute side effects were worse with concurrent CDDP, which may suggest why clinicians favored CDDP in younger, healthier patients.

One of the limitations of the SEER-Medicare analysis discussed above is the lack of complete HPV/p16 data recorded. Since the publication of this SEER-Medicare analysis, data from the two prospective randomized have been presented and published. The NRG Oncology RTOG 1016 trial was designed as a non-inferiority trial comparing concurrent CTX *vs* CDDP in HPV-positive oropharyngeal cancers [5]. The study findings demonstrated that when compared to concurrent CDDP, CTX did not meet criteria for non-inferiority as OS was significantly worse (*p =* 0.0163) with 5-year OS rates of 77.9% compared to 84.6% in the CDDP group. Five-year progression free-survival (PFS) was also inferior with concurrent CTX, 67.3% *vs* 78.4%. Additionally, the risk of locoregional failure in the CTX group was more than twice that of CDDP (HR 2.05; *p =* 0.0005). Overall toxicity outcomes were similar between both groups, with differing profiles. Acute side effects were greater in the CDDP group. There were however no significant differences in late grade 3-4 toxicities between the two groups.

The De-ESCALaTE HPV study was a similarly designed study to RTOG 1016 performed in Europe, randomizing HPV positive oropharyngeal carcinoma patients between concurrent CDDP *vs* CTX [6]. The findings mirrored that of RTOG 1016. Two-year OS (HR 5.0; 89.4% *vs* 97.5%) and 2-year recurrence rates (HR 3.4; 6.0% *vs* 16.1%) were inferior with concurrent CTX. Overall toxicities were found to be similar between the two groups.

In summary, the SEER-Medicare study and two recent randomized controlled trials comparing concurrent CDDP *vs* CTX (NRG RTOG 1016 and De-ESCALaTE) suggest significantly improved survival outcomes with concurrent CDDP-based CRT, even for the more favorable HPV-positive population. Whether these findings can be extrapolated to the HPV negative smoking driven head and neck cancer remains an open question. For the HPV positive population, the data overall supports the current National Comprehensive Cancer Network (NCCN) guidelines favoring CDDP in those who can tolerate it. CTX and carboplatin continue to play a role in head and neck cancer and based on the SEER-Medicare findings, carboplatin appears to be the second best option if CDDP is not tolerated. Future randomized trials comparing standard of care CDDP to additional agents including carboplatin or potentially immunotherapy are needed for this patient population.
